# Supplementary material for: Splice-Junction-Based Mapping of Alternative Isoforms in the Human Proteome
Source: Cell Rep. Author manuscript; Available in PMC 2020 Jan 15. (PMC6961840; doi:10.1016/j.celrep.2019.11.026)
Supplement: 2 [file NIHMS1546469-supplement-2.zip › DF1/PXD010154/Esophagus/Esophagus_32_FDXR_LVDPQEMLRLLGALLLC.pdf]

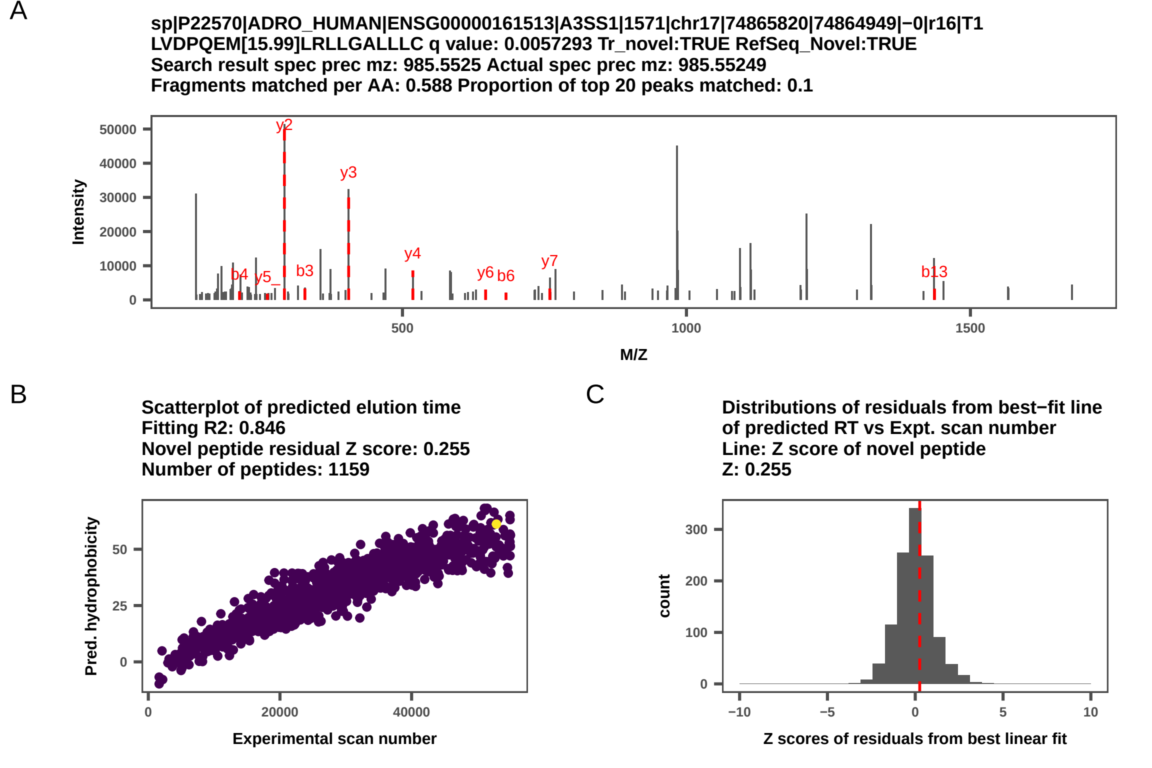
A
sp|P22570|ADRO_HUMAN|ENSG00000161513|A3SS1|1571|chr17|74865820|74864949|−0|r16|T1
LVDPQEM[15.99]LRLLGALLLC q value: 0.0057293 Tr_novel:TRUE RefSeq_Novel:TRUE
Search result spec prec mz: 985.5525 Actual spec prec mz: 985.55249
Fragments matched per AA: 0.588 Proportion of top 20 peaks matched: 0.1
Intensity
M/Z
y2
y3
y4
y7
b13
b4
y5_
b3
y6
b6
B
Scatterplot of predicted elution time
Fitting R2: 0.846
Novel peptide residual Z score: 0.255
Number of peptides: 1159
Pred. hydrophobicity
Experimental scan number
C
Distributions of residuals from best−fit line
of predicted RT vs Expt. scan number
Line: Z score of novel peptide
Z: 0.255
count
Z scores of residuals from best linear fit
